# Molecular characteristics of a potentially novel BVDV (*Pestivirus bovis*) genotype 1b isolate from commercial fetal bovine serum

**DOI:** 10.3389/fvets.2025.1629211

**Published:** 2025-09-24

**Authors:** Juanjuan Pan, Jianfeng Jiang, Shijiang Mi, Xintong Chen, Ruli Duan, Shandian Gao, Ling Kuang, Panpan Tong, Jinxin Xie

**Affiliations:** ^1^Laboratory of Animal Etiology and Epidemiology, College of Veterinary Medicine, Xinjiang Agricultural University, Urumqi, China; ^2^State Key Laboratory for Zoonotic Diseases, Key Laboratory for Zoonosis Research of the Ministry of Education, Institute of Zoonosis, College of Veterinary Medicine, Jilin Universitygrid, Changchun, China; ^3^Changchun Research Veterinary Institute, Chinese Academy of Agricultural Sciences, Changchun, China; ^4^State Key Laboratory for Animal Disease Control and Prevention, College of Veterinary Medicine, Lanzhou University, Lanzhou Veterinary Research Institute, Chinese Academy of Agricultural Sciences, Lanzhou, China; ^5^Xinjiang Key Laboratory of New Drug Research and Development for Herbivores, Urumqi, China

**Keywords:** *Pestivirus bovis*, genotype 1b, fetal bovine serum, contamination, novel strain

## Abstract

Bovine viral diarrhea virus (BVDV) is commonly detected in biological products such as tissues and serum. This study identified BVDV contamination in a commercially available fetal bovine serum (FBS) sample. To determine whether the detected virus was infectious or merely genetic material, the FBS was inoculated into Madin-Darby bovine kidney (MDBK) cells. Following six serial passages, both indirect immunofluorescence assay results and electron microscopic visualization of viral particles confirmed the presence of infectious BVDV. The isolated strain, designated BI-2023, had a complete genome length of 12,273 nucleotides. Comparative sequence analysis showed that the 5′ untranslated region (UTR) and full genome of BI-2023 shared 87.4–97.1% and 92.4–95.2% nucleotide identity, respectively, with reference strains of BVDV1 (*Pestivirus bovis*) genotype 1b. Phylogenetic analyses based on the 5′UTR and whole genome placed BI-2023 within the genotype 1b cluster of *Pestivirus bovis*. Several amino acid substitutions were identified in the E2 and E^rns^ proteins of the BI-2023 regions involved in immune evasion and viral secretion. This suggests this strain may represent a distinct variant within the genotype 1b group. These results highlight the critical need for routine viral screening in commercial FBS preparations.

## Introduction

Fetal bovine serum (FBS) is an essential supplement in cell culture media, widely used in manufacturing vaccines and other biologics. However, the adventitious agents, particularly viruses, can significantly compromise the safety and quality of these biological products (1–3). Bovine serum is known to potentially carry a variety of contaminating viruses, including bluetongue virus (BTV), adenovirus (BAV), poliovirus (BPV), respiratory syncytial virus (BRSV), viral diarrhea virus (BVDV), rabies virus (RV), parainfluenza virus type III (PIV III), and infectious bovine rhinotracheitis virus (IBRV), among others. BVDV is one of the most commonly detected contaminants in bovine serum samples (1–13).

The BVDV is a member of the *Flaviviridae* family and belongs to the genus *Pestivirus*. It is the primary causative agent of bovine viral diarrhea and mucosal disease in cattle, conditions characterized by clinical signs such as fever, diarrhea, decreased milk yield, and reproductive dysfunctions (14). BVDV is a single-stranded, positive-sense RNA virus with a genome approximately 12.3 kb in length (15). Globally, two major genotypes of BVDV have been identified: BVDV-1 and BVDV-2. According to the International Committee on Taxonomy of Viruses (ICTV), these are now classified as *Pestivirus bovis* (BVDV-1) and *Pestivirus tauri* (BVDV-2), respectively (15). Within *Pestivirus bovis*, at least 25 distinct genotypes (designated 1a to 1.25) have been recognized (16–22). Furthermore, BVDV exists in two biotypes: cytopathogenic (CP) and non-cytopathogenic (NCP), differentiated by their ability to induce cytopathic effects (CPE) in cultured cells.

In this study, a non-cytopathogenic strain of BVDV was successfully isolated from commercially available fetal bovine serum. Multiple amino acid substitutions were identified in the E2 and E^rns^ protein regions that play key roles in immune evasion and viral secretion. These mutations suggest the isolate may represent a novel BVDV1 (*Pestivirus bovis*) genotype 1b group variant. This discovery underscores the genetic diversity of circulating BVDV strains and highlights the critical need for routine surveillance to ensure the safety of bovine-derived biological materials.

## Materials and methods

### BVDV detection

Four commercial fetal bovine serum (FBS) samples originating from Israel were obtained from Biological Industries (LOT: 2153440) and ExCell Bio (LOT: 12B052). Viral RNA was extracted from 200 μL of each FBS sample using the Geneaid extraction kit, following the manufacturer’s protocol. Complementary DNA (cDNA) was synthesized using PrimeScript II Reverse Transcriptase (Takara, China) under the following thermal conditions: 65°C for 5 min, 42°C for 60 min, and 95°C for 5 min. The 5′-UTR of BVDV was then amplified by PCR using the primer pair 1F1 and 1R462 (Table 1). Amplification was performed with 2 × TransStart® FastPfu Fly PCR SuperMix (TransGen Biotech, Beijing, China) under the following cycling parameters: initial denaturation at 95°C for 2 min, followed by 35 cycles of denaturation at 95°C for 20 s, annealing at 52°C for 20 s, and extension at 72°C for 30 s, with a final extension step at 72°C for 5 min. The PCR products were analyzed via electrophoresis on a 1% agarose gel.

**Table 1:** Primers used in the present study.

Primer name	Sequence (5′-3′)	Product size (bp)
1F1	GTATACGAGGTTAGGCAAGTTCTCG	462
1R462	CTTGGTCGTATACTGGTTCCTCCAC	
2F469	GTAATCCTTTGTTTGGAGAAAGAGG	1,269
2R1737	GGTAGTGAGCTGTGTCTTGAATCAC	
3F1708	CTAGTGTGATTCAAGACACAGCTC	1,475
3R3182	AGAAGTCTCATCCACTTGTCTGTAG	
4F3131	CAAATGCAGACTGAAGAATGAGAG	1,645
4R4775	GTAGACTTCTTCCTCCTCATACCAG	
5F4722	CATAAAGTTAGGAACCAGACTGTAG	1,469
5R6189	CCACTCTTATGCTCTCAGAAAATC	
6F6164	CAGATTTTCTGAGAGCATAAGAGTG	1,469
6R7659	GTGTAGCGAATGCAGTTTCATG	
7F7641	GAAACTGCATTCGCTACACTAGTG	1,540
7R9180	CTGGTTTTATAGTCCCGATATCTTC	
8F9159	GATATCGGGACTATAAAACCAGTAC	1,581
8R10739	CCAATCATCACTGACATCTCTCTTC	
9F10715	GAAGAGAGATGTCAGTGATGATTGG	1,559
9R12273	TGGGGCTGTTAAGGGTTTTCCCTAG	

### Cell culture and virus isolation

Madin-Darby bovine kidney (MDBK) cells, confirmed to be free of BVDV by the PCR described above, were kindly provided by the laboratory of Tu Changchun. MDBK cells were exposed to BVDV-positive fetal bovine serum for 2 h at 37°C in a humidified incubator for viral inoculation with 5% CO_2_. Following incubation, the inoculum was removed, and the cells were cultured in Dulbecco’s Modified Eagle Medium (DMEM) supplemented with 2% BVDV-negative FBS. The cultures were maintained under these conditions for 96 h, after which the cells underwent three freeze–thaw cycles to release intracellular virus particles. Six additional cell passages were performed to enhance viral propagation. For electron microscopy, virus particles were purified via sucrose density gradient centrifugation and negatively stained using 2% phosphotungstic acid.

### Indirect immunofluorescence assay (IFA)

MDBK cells were seeded into 12 wells of a 96-well culture plate and allowed to grow until they reached 70–80% confluency. Four wells were inoculated with the previously prepared virus solution, while the remaining eight were negative controls. The cells were then incubated for 48 h. Following incubation, the cells were fixed with 4% formaldehyde for 30 min, then blocked with 1% bovine serum albumin (BSA) at room temperature for 30 min. A mouse monoclonal antibody targeting the BVDV E2 protein (IgG2a isotype; kindly provided by Prof. Changchun Tu) was applied at a 1:1000 dilution, and the plates were incubated for 1 h at 37°C. After washing with phosphate-buffered saline containing Tween 20 (PBST), the cells were incubated with Alexa Fluor 488-conjugated donkey anti-mouse IgG (Invitrogen) at a 1:500 dilution for 2 h at 37°C. Following a final wash with PBST, fluorescence was visualized using an Olympus CKX53 fluorescence microscope at 5 × magnification.

### Complete genomic amplification and sequencing

The PCR assays were established to amplify the BVDV genomic sequence using primer pairs detailed in Table 1. RNA extraction and reverse transcription-PCR (RT-PCR) were carried out following the same protocol described previously for BVDV detection. Positive PCR products were cloned into the pESI-T vector (Yeasen Biotech) and transformed into *E. coli* DH5α chemically competent cells (Weidi Biotech). Three independent clones from each amplicon were selected for Sanger sequencing (Sangon Biotech).

### Phylogenetic analyses

A total of 23 BVDV reference strains representing diverse genotypes from the United States, Japan, Germany, and China were retrieved from GenBank. Detailed information, including GenBank accession numbers, is presented in Figures 3, 4. Sequence similarity analyses were performed using MegAlign software (Lasergene v7.1). Phylogenetic trees were constructed using the maximum-likelihood method based on the Tamura–Nei model. To assess the robustness of the inferred tree topologies, bootstrap analysis with 1,000 replicates was conducted (23).

**Figure 2:**
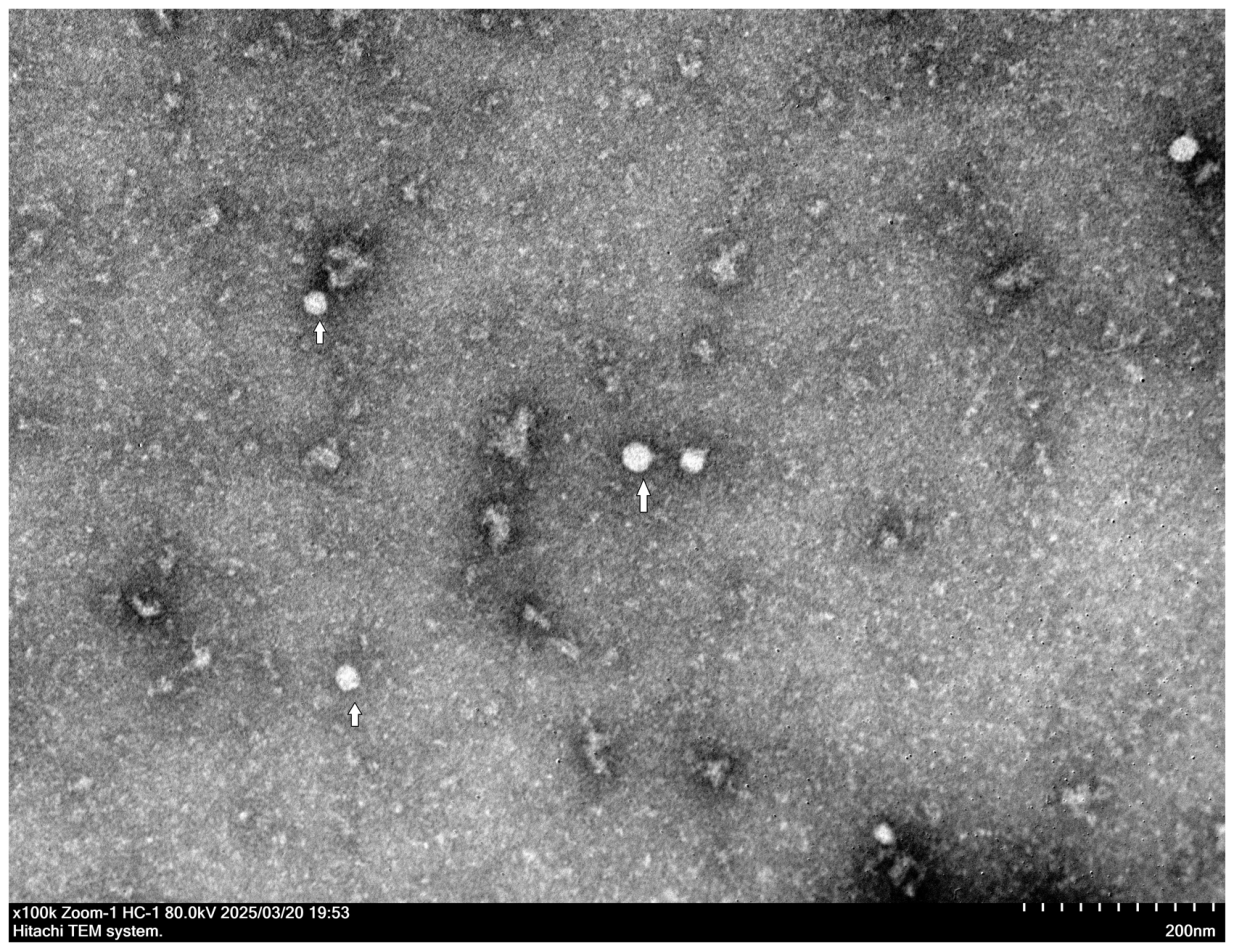
The virions of BVDV BI-2023 display distinct morphologies.

**Figure 3:**
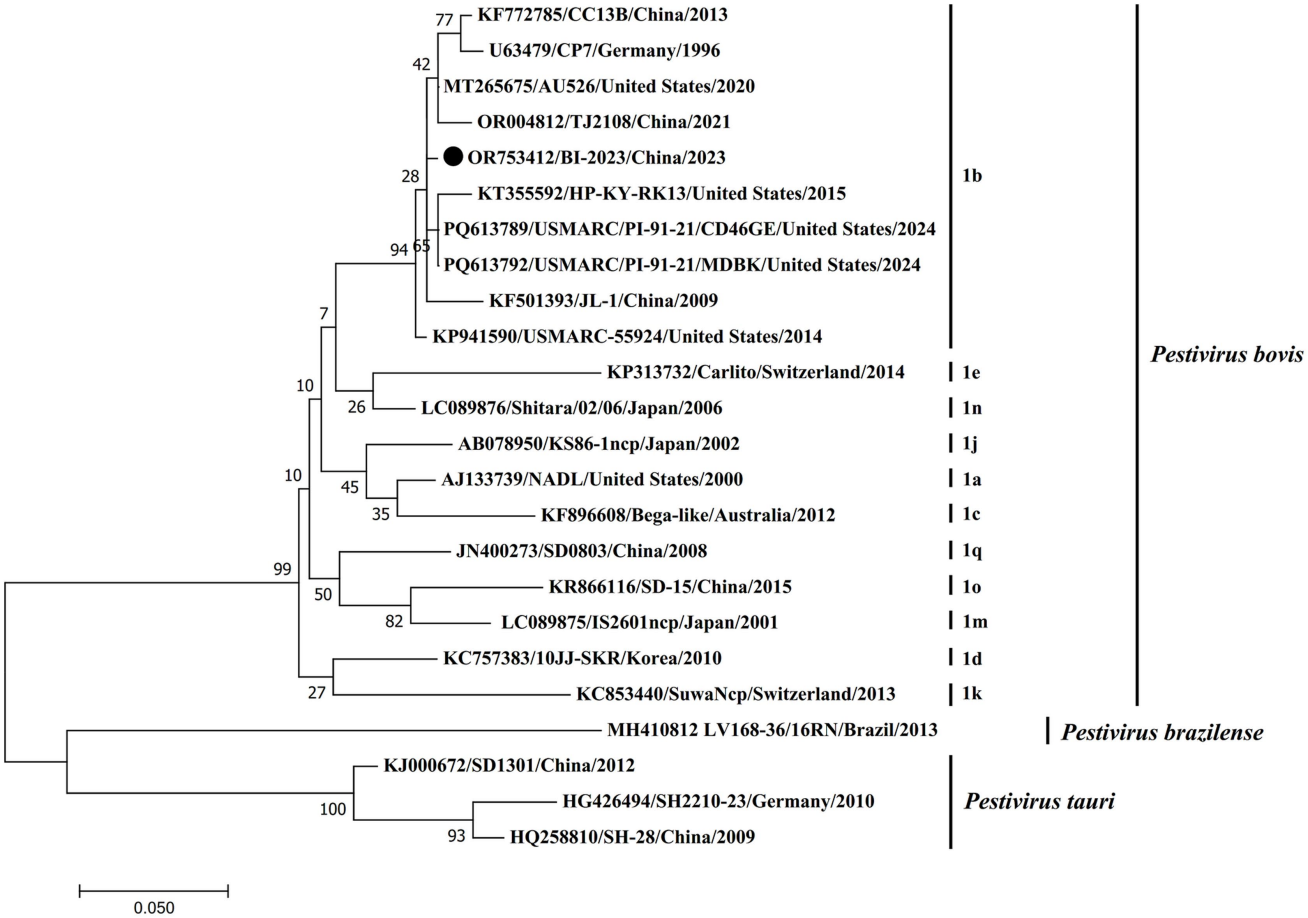
Phylogenetic analysis of the new isolates with 23 BVDV reference strains based on the 5′-UTR nucleotide sequences. The 5′-UTR nucleotide sequences of the new isolates were aligned with 23 reference BVDV strains, and phylogenetic analysis was carried out by the maximum-likelihood (ML) method using MEGA7 software (1,000 bootstrap replicates). Fixed circles represent the BVDV BI-2023 strain isolated in this study. The 5′-UTR nucleotide sequences of the reference BVDV strains were obtained from the GenBank data.

## Results

### Virus isolation and identification

**Figure 1:**
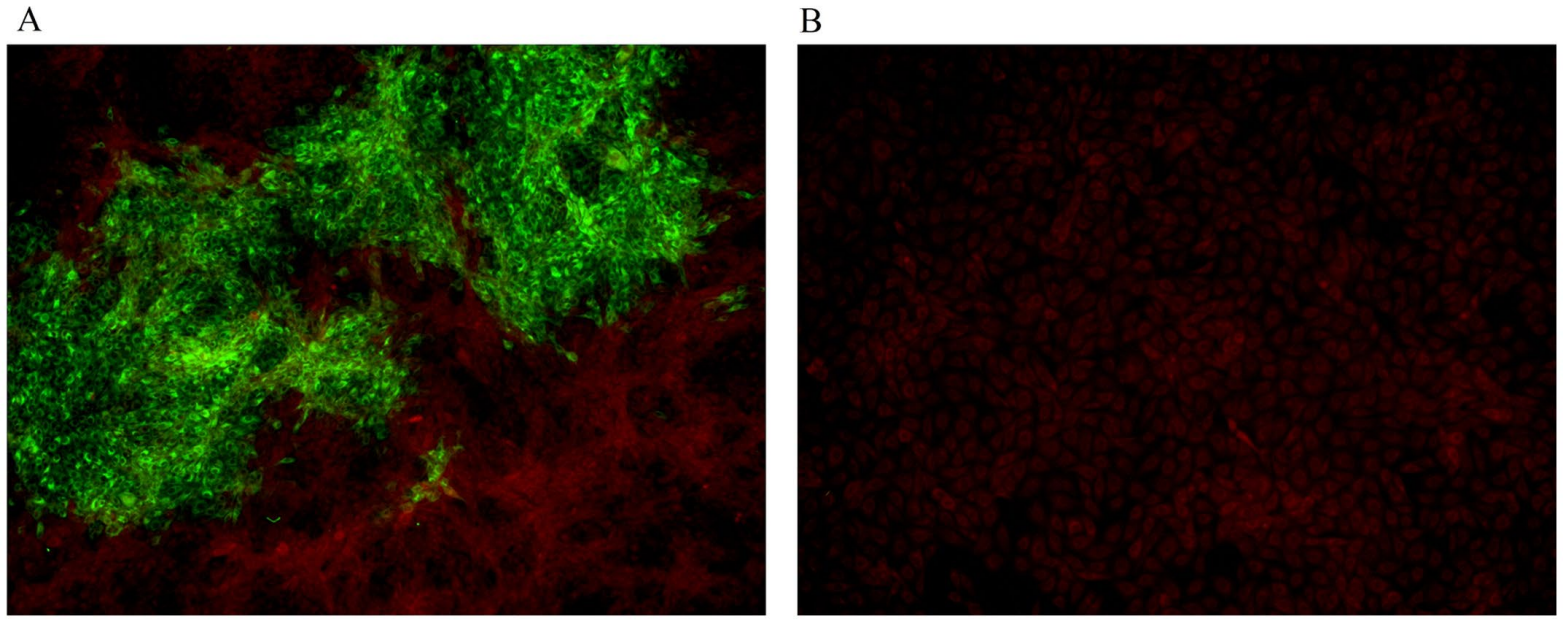
IFA for BVDV. **(A)** IFA detection of BVDV-infected MDBK cells using an anti-BVDV monoclonal antibody. **(B)** MDBK cells negative for BVDV were used as the control.

RT-PCR analysis revealed a specific 462 bp amplicon corresponding to BVDV in all FBS samples obtained from Biological Industries, but not in those from ExCell Bio. The two BVDV-positive commercial FBS was then used to inoculate MDBK cells, which were monitored daily. After six successive passages, no apparent cytopathic effects were observed in the infected cells. However, BVDV RNA was detected by RT-PCR in one of the two culture supernatant, indicating the presence of an NCP BVDV strain. This isolate was designated BVDV BI-2023. The presence of BVDV BI-2023 in the commercial FBS was further confirmed by indirect immunofluorescence assay (Figures 1A,B), and its identity was validated via transmission electron microscopy (TEM) (Figure 2).

### Sequence analyses of 5’-UTR

To determine the genotype of BVDV BI-2023, phylogenetic analysis was performed using the maximum likelihood method based on its 5’-UTR sequence, alongside 20 BVDV reference strains (Figure 3). The results showed that BVDV BI-2023 clustered within the BVDV1 (*Pestivirus bovis*) genotype 1b group. Sequence alignment revealed that the new isolate shared 87.4–97.1% nucleotide identity with known BVDV1 (*Pestivirus bovis*) genotype 1b strains, including USMARC/PI-91-21/CD46GE, USMARC/PI-91-21/MDBK, AU526, CP7, CC13B, and JL-1. However, its similarity with other BVDV1 (*Pestivirus bovis*) genotype ranged from 68.1 to 88.6% (Table 2). These findings support the classification of BVDV BI-2023 as a member of the BVDV1 (*Pestivirus bovis*) genotype 1b lineage.

**Table 2:** Homology analysis of the BVDV BI-2023 5’UTR gene in this study.

Isolate	Nucleotides (%)
BVDV1b CC13B, CP7, JL-1, and TJ2108 etc.	87.4–97.1
BVDV1q SD0803	83.8
BVDV1m SD-15	82.6
BVDV1o IS2601ncp	83.8
BVDV1n Shitara02/06	88.6
BVDV1k SuwaNcp	83.3
BVDV1d 10JJ-SKR	85.5
BVDV1c Bega-like	68.1
BVDV1j KS86-1ncp	85.3
BVDV1a NADL	86.7
BVDV1e Carlito	83.1
BVDV2a SH-28	72.0
BVDV2b SH2210-23	71.5
BVDV2c SD1301	71.0
BVDV3 LV168/36/16RN	67.6

### Whole-genome sequence comparisons and phylogenetic analyses

To investigate the molecular characteristics of the isolate, the full-length genome of BVDV BI-2023 was amplified and sequenced, yielding a sequence of 12,273 nucleotides, which has been submitted to GenBank under accession number OR753412. Comparative analysis revealed that BI-2023 shared 92.4–95.2% nucleotide identity with BVDV1 (*Pestivirus bovis*) genotype 1b reference strains (Table 3). In particular, it showed 95.2% identity with the BVDV1 (*Pestivirus bovis*) genotype 1b reference strain CP7, confirming its classification as a BVDV1 (*Pestivirus bovis*) genotype 1b group member (Table 3). Further multiple sequence alignments revealed that BVDV BI-2023 isolated in the present study shared 90.7–97.0% nucleotide (Table 4) and 90.1–100% amino acid (Table 5) identities for the N^pro^, Core, E^rns^, E1, E2, p7, NS2, NS3, NS4A, NS4B, NS5A, and NS5B genes, with BVDV1 (*Pestivirus bovis*) genotype 1b reference strain CC13B, CP7, JL-1, USMARC/PI-91-21/CD46GE, USMARC/PI-91-21/MDBK, and TJ2018 etc.

**Table 3:** Homology analysis of the full genome of BVDV BI-2023 in this study.

Isolate	Nucleotides (%)
BVDV1b CC13B, CP7, JL-1, and TJ2108 etc.	92.4–95.2
BVDV1q SD0803	79.0
BVDV1m SD-15	79.1
BVDV1o IS2601ncp	78.8
BVDV1n Shitara02/06	78.7
BVDV1k SuwaNcp	79.5
BVDV1d 10JJ-SKR	80.3
BVDV1c Bega-like	80.3
BVDV1j KS86-1ncp	80.4
BVDV1a NADL	79.7
BVDV1e Carlito	79.8
BVDV2a SH-28	70.1
BVDV2b SH2210-23	70.1
BVDV2c SD1301	69.8
BVDV3 LV168/36/16RN	67.6

**Table 4:** Homology analysis of the BVDV BI-2023 and BVDV1 (*Pestivirus bovis*) genotype 1b reference strains 11 genes in this study.

Gene	Nucleotides (%)
*N^pro^*	91.5–97.0
*Core*	90.7–93.6
*E^rns^*	92.4–95.9
*E1*	91.3–94.4
*E2*	90.5–93.8
*p7*	91.0–93.3
*NS2*-*NS3*	94.1–96.6
*NS4A*	93.4–97.0
*NS4B*	91.7–95.2
*NS5A*	90.7–94.1
*NS5B*	93.6–95.4

**Table 5:** Homology analysis of the BVDV BI-2023 and BVDV1 (*Pestivirus bovis*) genotype 1b reference strains 11 proteins in this study.

Protein	Amino acid (%)
N^pro^	94.6–97.0
Core	92.3–95.2
E^rns^	96.5–98.7
E1	93.3–96.4
E2	90.1–92.2
p7	91.4–94.3
NS2-NS3	96.4–98.0
NS4A	100
NS4B	95.6–96.8
NS5A	91.3–95.4
NS5B	96.4–97.4

**Figure 4:**
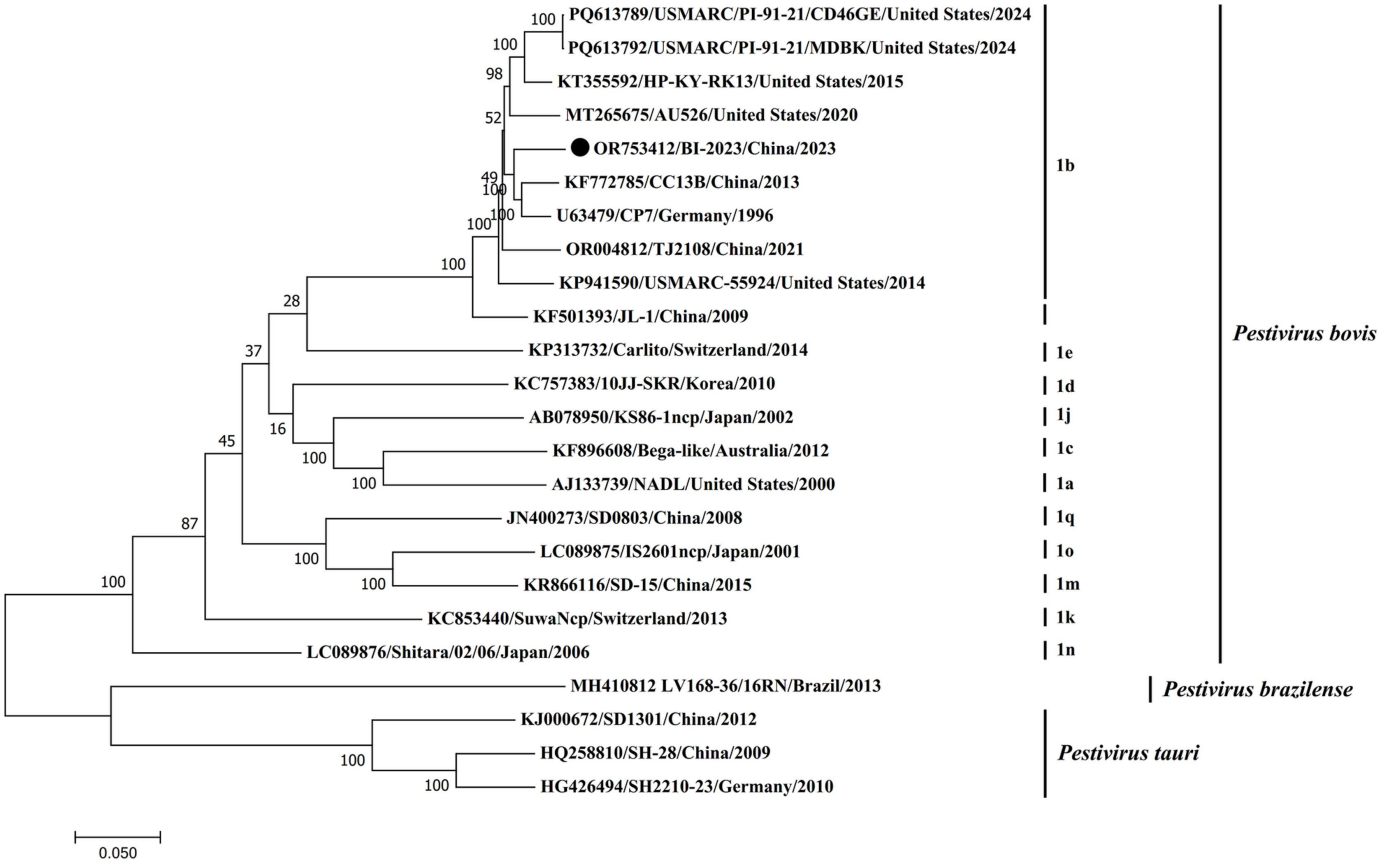
Phylogenetic analysis of the new isolates with pestiviruses based on the full-length nucleotide sequences. The genomic sequences of the new isolates with pestiviruses were based on the full-length nucleotide sequences. The genomic sequences of the new isolates were generated and aligned with representative pestiviruses, including *Pestivirus bovis*, *Pestivirus tauri,* and *Pestivirus brazilense,* in this study. Phylogenetic analysis was carried out using the maximum-likelihood (ML) method using MEGA7 software (1,000 bootstrap replicates). Fixed circles represent the BVDV BI-2023 strain isolated in this study.

Furthermore, phylogenetic analysis based on the complete genome sequence demonstrated that BVDV BI-2023 is closely aligned with established BVDV1 (*Pestivirus bovis*) genotype 1b reference strains, including CC13B, CP7, JL-1, USMARC/PI-91-21/CD46GE, and TJ2018. As depicted in Figure 4, BI-2023 clusters within the genotype 1b lineage, highlighting its strong genetic relatedness to these strains and further supporting its classification as a member of BVDV1 (*Pestivirus bovis*) genotype 1b (Figure 4).

### BVDV BI-2023 encodes E2, and E^rns^ has several unique mutations in functional domains

**Figure 5:**
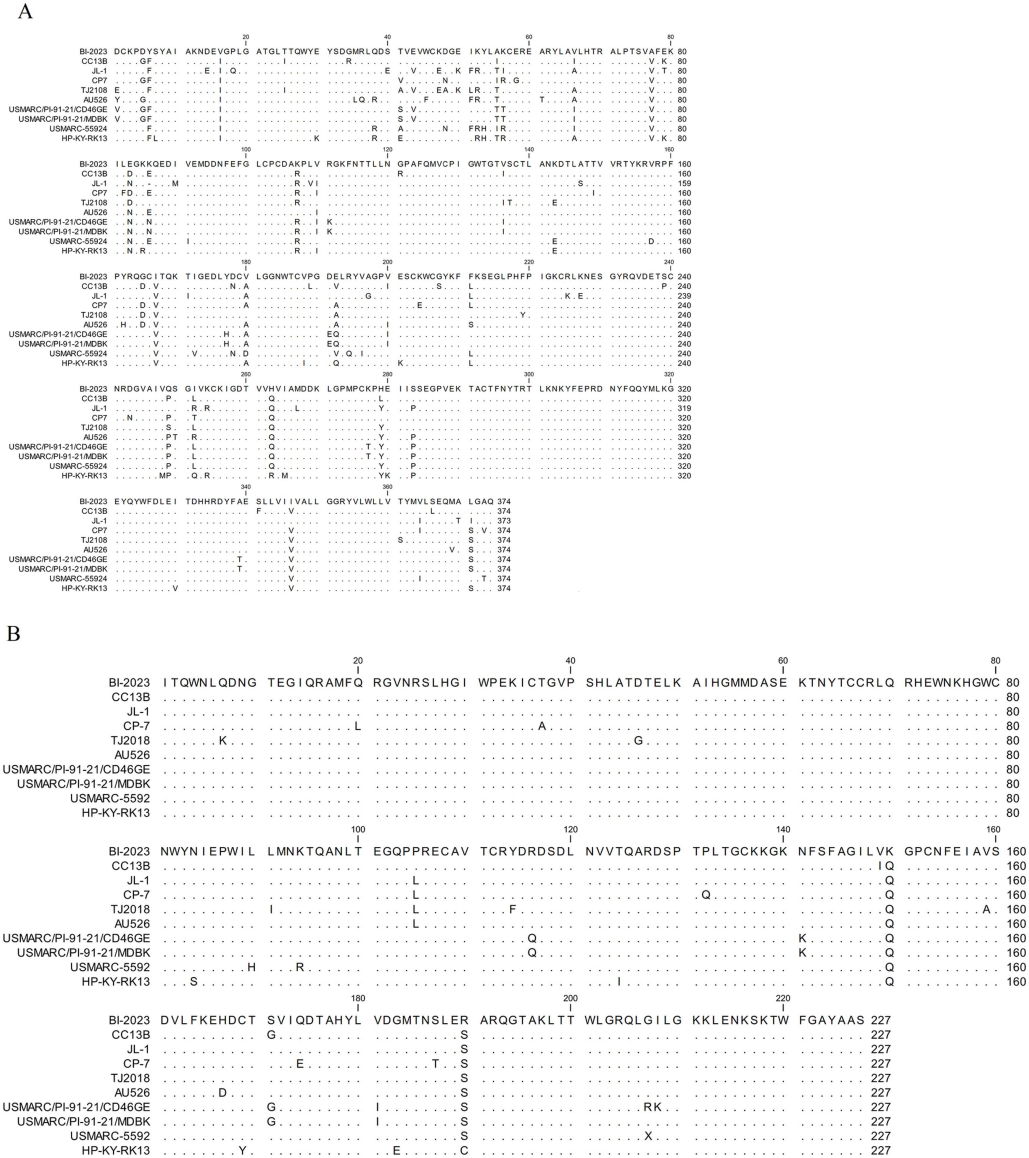
Analysis of the E2 and E^rns^ protein sequence. Analysis of the BVDV1b BI-2023 E2 protein sequence **(A)**. Analysis of the BVDV1b BI-2023 E^rns^ protein sequence **(B)**. Sequence identity with the BVDV1b BI-2023 isolate (CLC Sequence Viewer 8) is indicated by dots.

A comparative analysis of the E2 and E^rns^ amino acid sequences among BVDV1 (*Pestivirus bovis*) genotype 1b strains revealed distinct differences in BVDV BI-2023. Residues F6, I16, I/T55, V77, and D/N83 within domain DA, as well as L/T/R252 and O263 in domain DC of the E2 protein, were conserved across all analyzed BVDV1 (*Pestivirus bovis*) genotype 1b reference strains except in BVDV BI-2023 (Figure 5A). Furthermore, two unique substitutions were identified in the E^rns^ protein: Q150K within one of the seven known linear epitopes, and S190R in the C-terminal domain. These findings suggest that BVDV BI-2023 may represent a novel variant within the BVDV1 (*Pestivirus bovis*) genotype 1b group (Figure 5B).

## Discussion

Bovine serum has been an essential component in cell culture systems for over five decades, yet it continues to be frequently contaminated with adventitious viruses. There are numerous viral agents in bovine serum and its derivatives, with BVDV being the most commonly detected contaminant (1–13). In this study, an NCP strain of BVDV was successfully detected and isolated from commercial FBS. However, this analysis did not extend to other possible adventitious agents or pathogens, which may also pose significant risks to the safety of biological products. This highlights the critical importance of thorough screening for viral contaminants in commercial serum and related biological materials before using.

While PCR offers specific and sensitive detection for known viruses, it may fail to identify uncharacterized variants or closely related viral species. Commercial serum products may harbor undetected contaminants without comprehensive testing, adversely affecting experimental reliability. NCP-type BVDV, in particular, can persistently infect cultured cells without producing overt cytopathic effects, making it difficult to detect and potentially compromising experimental results, diagnostic assays, and clinical trial outcomes. Advanced techniques such as next-generation sequencing provide a more robust approach for identifying these hidden viral contaminants.

Based on 5′-UTR sequence comparisons, BVDV strains are classified into two species: *Pestivirus bovis* (BVDV-1) and *Pestivirus tauri* (BVDV-2). *Pestivirus bovis* has been subdivided into 25 genotypes (1a to 1.25) (16–22). Previous unpublished sequence data have reported the presence of BVDV1 (*Pestivirus bovis*) genotype 1b in various regions of Israel (24). In the current study, a putative BVDV1 (*Pestivirus bovis*) genotype 1b strain was identified in commercial FBS sourced from Israel, suggesting that this virus may be circulating within Israeli cattle populations. Further investigations are warranted to screen additional batches or sources of FBS to assess the extent of viral contamination.

The FBS is an essential raw material for cell culture applications (1–3). However, if contaminated with viruses, it can transmit these pathogens into cell culture-derived vaccines and other biological products (1–3). Previous studies have demonstrated that commercial bovine serum products from various regions in China frequently contain at least one type of viral contaminant (4–9). In a 1991 investigation, the National Animal Disease Center identified 93 viral isolates from 190 batches of commercial FBS (5). Multiple reports have also documented the presence of BVDV1 in imported commercial FBS, highlighting the widespread issue of BVDV contamination in both domestic and imported serum sources (4, 6–9).

Furthermore, a novel putative pestivirus species provisionally named “HoBi-like” and currently classified by the International Committee on Taxonomy of Viruses as *Pestivirus brazilense* was initially detected in Europe in FBS imported from Brazil (25, 26). Contaminated serum can interfere with the diagnosis of viral infection, and using such contaminated serum to produce vaccines can lead to seroconversion or illness in vaccinated animals (4–6). These findings underscore the frequent occurrence of BVDV contamination in cattle-derived materials.

Currently, China restricts the import of FBS to countries certified free of bovine spongiform encephalopathy, such as Australia, New Zealand, and Uruguay. However, the circulation of American FBS with unverified origins in the domestic market raises concerns about the effectiveness of animal disease prevention and control efforts in China (6). In this study, the BVDV BI-2023 strain identified in the FBS was an NCP type, posing a potential risk of widespread infection and the birth of persistently infected animals. As a result, laboratories must screen FBS for exogenous viruses and determine their genotypes before use.

Genome sequencing of the BVDV BI-2023 strain revealed a total of 12,273 nucleotides encoding a polyprotein comprising four structural and seven non-structural proteins. Based on 5’-UTR sequence comparison, this isolate was classified as a BVDV1b genotype. A previous study by Zhu et al. proposed that the dominant BVDV1 (*Pestivirus bovis*) genotype 1b strains circulating in China (e.g., CC13B and JL-1) likely originated from Europe (27). In the present study, alignment analyses using the full genome, 5’-UTR, structural, and non-structural protein sequences showed that BVDV BI-2023 shared high sequence identity with several foreign reference strains, including USMARC/PI-91-21/CD46GE, USMARC/PI-91-21/MDBK, and AU526, all originally isolated in the United States. Phylogenetic analysis of the full genome and the 5’-UTR further confirmed a closer genetic relationship between BVDV BI-2023 and these United States-derived strains than with other BVDV1 (*Pestivirus bovis*) genotype 1b isolates. These findings support the hypothesis that the BVDV1 (*Pestivirus bovis*) genotype 1b strains currently reported in China may have been introduced abroad, highlighting the need to monitor this genotype in imported FBS and domestic cattle populations.

Glycoprotein E2 is the primary envelope protein displayed on the surface of BVDV virions and plays a key role in eliciting neutralizing antibody responses during infection (28). Structurally, the E2 protein is organized into four domains: DA (residues 4–87), DB (residues 88–164), DC (residues 165–271), and DD (residues 272–333) (28). There are three immunodominant regions in the BVDV E2 protein, located at residues 16–26, 71–74, and 142–150 (29–31), all of which are within the DC domain and contribute to neutralization. In this study, a comparison of BVDV BI-2023 with other BVDV1 (*Pestivirus bovis*) genotype 1b reference strains indicated that the BVDV BI-2023 E2 protein had one unique mutations located at residue I16V of immunodominant regions (Figure 5A). The unique mutation may reduce antibody-binding affinity, potentially enabling BVDV BI-2023 to evade host immune recognition.

E^rns^ is another envelope glycoprotein involved in virus neutralization and is distinguished by its intrinsic ribonuclease (RNase) activity (28). Seven linear epitopes of the E^rns^ protein from BVDV1 (*Pestivirus bovis*) genotype 1b reference strains ^31^GIWPEKIC^38^, ^65^NYTCCKLQ^72^, ^127^QARNRPTT^134^, ^145^SFAGTVIE^152^, ^161^VEDILY^166^, ^114^CRYDKNTDVNV^124^, and ^116^YDKNTDVNV^124^ have been identified as key antibody-interacting regions (28). In the current study, BVDV BI-2023 showed a unique mutation within the fourth linear epitope, altering 145SFAGTVIE152 to 145SFAGTVQ151KE152 (Figure 5B), implying potential differences in antibody-binding properties between BVDV BI-2023 and reference genotype 1b strains. Furthermore, the C-terminal region of E^rns^ is critical for its secretion and intracellular retention, with residues 183, 190, and 208 playing important roles (28). Sequence analysis revealed a unique R190S substitution in BVDV BI-2023 (Figure 5B), suggesting that this mutation might affect the protein’s secretion dynamics. These findings indicate that BVDV BI-2023 shows molecular differences in E^rns^ compared to established genotype 1b reference strains.

## Conclusion

In conclusion, this study identified a potentially novel strain of BVDV1 (*Pestivirus bovis*) genotype 1b from commercially imported FBS. Given the risk of viral contamination in cell cultures and biological products and the potential for broader dissemination, the implicated serum batch should be discarded. These findings highlight the urgent need for regulatory authorities to implement routine viral screening protocols for all imported serum batches to safeguard laboratory research and animal health.

## Data Availability

The datasets presented in this study can be found in online repositories. The names of the repository/repositories and accession number(s) can be found in the article/supplementary material.
